# Effectiveness of Bundled Interventions for the Prevention of Neonatal Hypothermia in Low-Income Settings: A Quality Improvement Project in a Referral Hospital in Ethiopia

**DOI:** 10.3390/children12060709

**Published:** 2025-05-30

**Authors:** Margherita Baracetti, Eleni Hagos, Jiksa Tolera, Francesco Cavallin, Enzo Facci, Giovanni Putoto, Fabio Manenti, Daniele Trevisanuto, Andrea Pietravalle

**Affiliations:** 1 Doctors with Africa CUAMM, Wolisso P.O. Box 250, Ethiopia; margheritabaracetti@gmail.com (M.B.); e.hagos@cuamm.org (E.H.); e.facci@cuamm.org (E.F.); 2 St. Luke Wolisso Hospital, Wolisso P.O. Box 250, Ethiopia; jiksatolera@gmail.com; 3 Independent Statistician, 36020 Solagna, Italy; cescocava@libero.it; 4 Doctors with Africa CUAMM, 35121 Padua, Italy; g.putoto@cuamm.org (G.P.); f.manenti@cuamm.org (F.M.); 5 Department of Woman’s and Child’s Health, University of Padua, 35122 Padua, Italy; daniele.trevisanuto@unipd.it

**Keywords:** low- and middle-income countries, neonatal hypothermia, quality improvement

## Abstract

Background: Hypothermia at admission and in the following days is a major risk factor for neonatal mortality in both high- and low-resource settings. Implementing hypothermia prevention procedures is not an easy goal to achieve, and the few studies currently available in low-income countries focus mainly on temperature at admission. Deviation from normothermia does not exhaust its effects upon admission, with a demonstrated negative impact of hypothermia also during the first days of life. Objective: The aim of this study was to evaluate the effectiveness of bundled interventions in preventing neonatal hypothermia at admission and during hospitalization in a low-resource setting. Methods: This was a retrospective, observational, before–after study comparing a pre- (December 2023–February 2024) and a post-quality improvement intervention period (April–June 2024). The outcome measures included admission temperature, average temperature during hospitalization, number of hypothermia episodes, temperature checks per day during hospitalization, and mortality. Results: From the pre- to the post-intervention period, the median admission temperature increased from 35.6 °C to 36.0 °C (*p* = 0.004). Median temperature during hospitalization increased from 36.3 °C to 36.7 °C (*p* < 0.0001). Mild and moderate hypothermia episodes decreased from 1.0 to 0.5 and from 0.7 to 0.2 episodes per day (*p* < 0.0001). Conclusions: In a Sub-Saharan referral hospital, the implementation of bundled interventions to maintain the warm chain improved neonatal temperature at admission and during hospitalization, and reduced hypothermia episodes during hospitalization.

## 1. Introduction

Maintaining normothermia represents a major challenge for newborn survival, and hypothermia remains an independent predictor of neonatal morbidity and mortality in both high- and low-resource countries [1]. Sustaining a temperature between 36.5 and 37.5 °C after birth through admission and stabilization has been associated with the lowest rates of adverse outcomes [2]. Several quality improvement studies have demonstrated the impact of a bundle of strategies to prevent heat loss in high-income countries (HICs) [1]. Figures from Sub-Saharan Africa report a high prevalence of newborn hypothermia ranging 44–85%, with an associated mortality rate reaching peaks of 94.9% in hospital-based studies [3]. Recent evidence from low-income countries (LICs) confirmed the relationship between normothermia at admission and reduced risk of mortality [4].

Deviation from normothermia does not exhaust its effects upon admission, with a demonstrated negative impact of hypothermia also during the first day of life [5]. This underlines the importance of thermal care during an extended period after birth. The prevention of hypothermia in low-resource settings must focus on simple and effective interventions to maintain the warm chain, which is a set of 10 interlinked procedures that should be implemented at birth and during the following hours and days (including (1) warm delivery room, (2) immediate drying, (3) skin-to-skin contact, (4) breastfeeding, (5) bathing and weighing postponed, (6) appropriate clothing/bedding, (7) mother and baby together, (8) warm transportation, (9) warm resuscitation, and (10) training/awareness raising) [6].

To date, most evidence on the prevention of hypothermia in newborns comes from studies conducted mainly in middle-/high-income countries and focused on interventions in the delivery room to improve temperature at admission in preterm newborns (through the use of a plastic bag, a plastic cap, a thermal mattress, and heated humidified respiratory gas) [7].

A few studies from upper-middle- and middle–high-income countries have shown the effectiveness of bundled interventions to decrease the incidence of neonatal hypothermia [8,9,10,11]. These interventions were carried out using Plan–Do–Study–Act (PDSA) cycles and focused on reducing hypothermia at admission through strengthening 1 or more of the 10 steps of the warm chain.

To date, only one study has investigated the implementation of a thermoregulation bundle in low-income countries [12].

## 2. Aim

The aim of the present study was to evaluate the effectiveness of bundled interventions in preventing neonatal hypothermia at admission and during hospitalization in a low-resource setting.

## 3. Materials and Methods

### 3.1. Study Design

**Figure 1:**
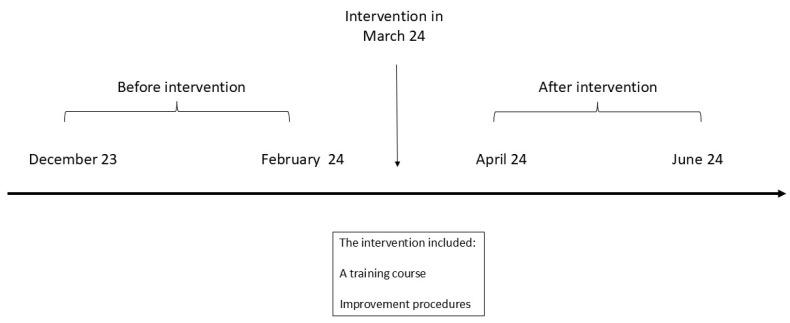
Timeline of the intervention and definition of the study periods. Details on the intervention are reported in Table 1.

This retrospective, observational, before–after study evaluated the effectiveness of bundled interventions in preventing neonatal hypothermia in a low-resource setting in Sub-Saharan Africa. The study compared a pre-intervention period (December 2023–February 2024) and a post-intervention period (April–June 2024) (Figure 1).

### 3.2. Setting

This study was performed at the St. Luke Catholic Hospital and College of Nursing and Midwifery in Wolisso, Ethiopia. This is the referral hospital of the South West Shoa Zone, Oromiya region, which has a population of about 1.1 million inhabitants. It accounts for around 3500 deliveries every year. The Neonatal Special Care Unit (NSCU) has 900 admissions per year and is equipped with 16 beds. The nurse/patient ratio is 1 to 5 and the doctor/patient ratio 1 to 8 per shift [13].

### 3.3. Patients

All infants who were admitted to the NSCU and Kangaroo Mother Care (KMC) room in the study period were eligible for inclusion in the study. Outborn infants and those admitted with fever were excluded.

### 3.4. Intervention

In March 2024, a structured process focused on hypothermia prevention was supported and implemented by the Italian NGO “Doctors with Africa Cuamm”, that acts from 1950 to protect and improve the wellbeing and health of vulnerable communities in Sub-Saharan Africa through long-term healthcare cooperation projects [14]. The bundle interventions included training activities for the healthcare staff and improved procedures in the delivery room, NSCU, and KMC room. The project core team consisted of the local neonatologist, the head nurse, and the pediatric resident. In addition to monitoring and supervising the work of the healthcare staff, the team was responsible for actively involving and training caregivers.

The training activity dedicated to the staff of the delivery room (midwives), of the NSCU (nurses) and to the students of both categories, was carried out through a series of theoretical lessons that were held over a period of 1 week. The 10 steps of the "warm chain" represented the focus of the training process, with particular attention to the extension of preventive measures also in the phase following management in the delivery room. The training of caregivers mainly involved the mothers of newborns admitted to the KMC room, with specific attention to the correct execution of the KMC and its benefits.

The interventions implemented in the delivery room focused mainly on improving the infrastructure and equipment in association with the redefinition and strengthening of specific procedures: to ensure an adequate room temperature at the time of delivery, an electric radiator was positioned in the delivery room and turned on at least 1 hour before delivery; to limit heat loss by convection, a door was positioned at newborn perinatal assistance’s room; to manage the newborn in a warm environment and provide heat by radiation in the immediate postpartum phase, it was recommended to turn on the radiant warmer at least half an hour before delivery with 100% heater output; to avoid heat loss by evaporation, the indications to remove wet linens and dry the newborn with a prewarmed towel immediately after birth were strengthened; To maintain normothermia in the first hour of life, skin-to-skin contact was recommended, ensuring heat transfer by conduction; Indications for early breastfeeding have been stressed to ensure adequate energy support for thermogenesis; To ensure normothermia during the transfer from the delivery room to the ward, instructions were given to wrap the newborn in pre-warmed cotton and cover him with wool blankets.

In the NSCU and KMC room, improvements to equipment and reinforcement of procedures were undertaken: to ensure an adequate environmental temperature an electric radiator was positioned and turned on if room temperature < 26 °C with care to keep doors and windows closed; to avoid heat loss by radiation, convection an d evaporation instructions were given to cover all newborns with cap, socks and also mittens in case of birth weight < 1500 g, to add a blanket for all the babies that are not under the radiant warmer and change the diaper at least 3 times a day; for unstable infants requiring management under a radiant warmer, advice has been given to keep it always on and to adjust the power % to ensure a body temperature between 36.5 and 37.5 °C; To overcome the need to keep the baby unclothed during phototherapy, instructions have been given to place two electric warmers near the cradle during the procedure; Specific guidance has been provided to encourage Kangaroo care not only in the KMC room but also for unstable newborns in the NSCU; Precise instructions were provided for close monitoring of temperature, especially after an episode of hypothermia, stressing the importance of reporting the data on vital sign’s charts and alert the next shift.

Details about the bundle interventions for each area are summarized in Table 1.

### 3.5. Outcome Measures

The primary outcome measures were the admission temperature and the average temperature during the hospitalization. The admission temperature was evaluated as a continuous variable and proportion of hypothermic neonates (<36.5 °C). The secondary outcome measures included the number of hypothermia episodes per day during the hospitalization, the number of temperature checks per day during the hospitalization, and mortality. The hypothermia episodes were stratified in mild (36.4–36 °C), moderate (32–35.9 °C), and severe hypothermia (<32 °C) episodes.

### 3.6. Data Collection

All data were retrospectively and anonymously collected from the hospital charts. The data collector could not be masked to the intervention period. Data collection included neonatal characteristics (sex and birth weight), mode of delivery, main diagnoses at admission (respiratory distress, jaundice, early onset infections, and perinatal asphyxia), oxygen therapy, duration of hospitalization, and information on the outcome measures.

### 3.7. Statistical Analysis

Categorical data were summarized as frequency and percentage, and numerical data as median and interquartile range (IQR). Comparisons between pre- and post-intervention periods were made using the Chi Square test and Fisher’s exact test (categorical variables), or the Mann–Whitney test (numerical variables). Statistically significance was set at 5%. Statistical analysis was carried out with R 4.4 (R Foundation for Statistical Computing, Vienna, Austria) [15].

## 4. Results

The analysis included 117 neonates in the pre-intervention period and 87 neonates in the post-intervention period. Neonatal characteristics at admission were not statistically different between the two periods (Table 2).

Table 3 summarizes the outcome measures in the pre- and post-intervention periods. The median temperature at admission increased from 35.6 °C (IQR 35.1–36.2) in the pre-intervention period to 36.0 °C (IQR 35.4–36.6) in the post-intervention period (*p* = 0.004). The proportion of hypothermia at admission was 80% in the pre-intervention period and 70% in the post-intervention period (*p* = 0.13).

The median duration of hospitalization was 3 days (IQR 2–5) in the pre-intervention period and 3 days (IQR 2–6) in the post-intervention period (*p* = 0.94).

The median temperature during hospitalization also increased from 36.3 °C (IQR 36.0–36.4) in the pre-intervention period to 36.7 °C (IQR 35.6–36.8) in the post-intervention period (*p* < 0.0001).

The number of mild and moderate hypothermia episodes per day decreased from the pre-intervention to the post-intervention period, while no episodes of severe hypothermia occurred (Table 3).

The healthcare staff performed a median of three temperature checks per day both in the pre- and post-intervention periods (*p* = 0.17) (Table 3).

Mortality rate was 5/117 (4%) in the pre-intervention period and 6/87 (7%) in the post-intervention period (*p* = 0.61) (Table 3).

## 5. Discussion

In a Sub-Saharan referral hospital, the implementation of bundled interventions to maintain the warm chain improved neonatal temperature at admission and during hospitalization, and reduced hypothermia episodes during hospitalization.

The World Health Organization (WHO) defines neonatal hypothermia as a core temperature below 36.5 °C, identifying categories of mild (36–36.4 °C), moderate (32–35.9 °C), or severe (<32 °C) hypothermia [16]. It is widely demonstrated that neonatal hypothermia at the time of admission has significant prognostic implications, and several multicenter observational studies showed an inverse relationship between admission temperatures and in-hospital mortality [2,17,18,19,20,21,22,23]. Birth weight and the degree of prematurity influence the severity of adverse outcomes, with a 28% increase in mortality and 11% increase in late-onset sepsis per 1 °C decrease in low-birth-weight neonates (BW < 2500 g) [23].

Furthermore, neonatal hypothermia triggers a chain reaction of physiological disturbances that leads to several complications including metabolic acidosis, jaundice, respiratory distress, hypoglycemia, and pulmonary hemorrhage [3].

A recent systematic review including randomized and quasi-randomized clinical trials of thermal care interventions in the delivery room for preterm newborns (through the use of a plastic bag, a plastic cap, a thermal mattress and heated humidified respiratory gas) showed an improvement in core body temperature with moderate certainty of evidence [7]. Of the 34 included studies, only 1 was conducted in a low-resource country, with the aim of comparing standard thermoregulation care (a blanket or radiant warmer) to standard thermoregulation care plus placement inside a plastic bag at birth [7].

A few studies from upper-middle- and middle–high-income countries have implemented a quality improvement (QI) approach with bundled interventions to improve thermal care for newborns prior to their admission in the Neonatal Intensive Care Unit (NICU) [8,9,10,11]. Based on the WHO Point-of-Care Quality Improvement Model [24], potential contributors to neonatal hypothermia have been identified, including staff awareness, environmental factors, and supply issues in the labor room, and challenges with rapidly and safely transferring sick newborns to the NICU. Subsequently, Plan–Do–Study–Act (PDSA) cycles were put in place to test and adapt possible solutions to these contributing factors. All studies finally observed a significant reduction in the proportion of hypothermic neonates at admission. Of note, only one study with a similar approach has been conducted in a low-resource country and yielded similar results [12].

Beyond the moment of admission, neonatal temperature during the first days of life has also been recognized as an important prognostic factor [25]. A recent study showed a high proportion of relevant thermal deviations during the first days of life occurring in newborns with normothermia at admission, with an increased likelihood of mortality in the case of becoming cold or hyperthermic on day 1 [5]. The NGO “Doctors with Africa Cuamm” supports the St. Luke hospital in Wolisso through a long-term project with the aim of improving the quality of pediatric and neonatal care. In supporting the management of the main clinical problems, Cuamm follows the WHO quality improvement models.

In the present study, we retrospectively investigated the impact of bundled interventions introduced into clinical practice to reduce neonatal hypothermia. In collaboration with the local staff, possible contributors of neonatal hypothermia were identified, and possible solutions were discussed and implemented in a bundle of interventions. The project core team consisted of the local neonatologist, the head nurse, and the pediatric resident. In addition to monitoring and supervising the work of the healthcare staff, the team was responsible for actively involving and training caregivers. The training activities and procedures were designed to reinforce the 10 steps of the warm chain not only in the delivery room but also during hospitalization in the NSC unit and KMC room (Table 1).

The quality improvement project started implementing the warm chain point 10, “training/awareness raising”, by delivering training on the importance of and actions for preventing neonatal hypothermia to delivery room staff, NSCU staff, and even students attending the ward. Similarly, information on the importance and procedures of KMC was communicated to mothers hosted in the Kangaroo room.

The delivery room improvements, developed to fulfill the warm chain point 1, “warm delivery room”, comprised placing a door at the newborn perinatal assistance room; placing an electric radiator in the delivery room and turning it on at least 1 h before delivery; and setting the radiant warmer on for at least half an hour before delivery with 100% heater output.

Warm chain point 2, “immediate drying”, was implemented by strengthening the procedures of removing wet linens after putting the newborn under the radiant warmer and wrapping them in prewarmed towels. Points 3, “skin-to-skin contact”, and 4, “breastfeeding”, were recommended in the first hour after birth.

Point 8, “warm transportation”, was reached by wrapping newborns with prewarmed cotton and swaddle covering with wool blankets before moving to NSCU.

The interventions implemented in the post-delivery phase had the following as their main focus:

Point 1, “warm delivery room”, extended to the NSCU and in the KMC room by turning on the electric radiator if room temperature < 26 °C, keeping doors and windows closed, putting two electric warmers near the cradle during phototherapy, and letting the radiant warmer always be on and setting the power % to ensure a body temperature between 36.5 and 37.5 °C.

Point 6, “appropriate clothing/bedding”, with warm clothes including cap, socks, mittens, and sweater.

Point 7, “mother and baby together”, by encouraging Kangaroo care in NSCU also for unstable babies and in the KMC room for all babies under 2000 g.

Our results showed a reduction from 80% to 70% in the proportion of hypothermic neonates at the time of admission to NSCU. Nonetheless, the temperature at admission indicated a clinically non-optimal transition from moderate to mild hypothermia, suggesting the need for further improvements. We believe that the modest impact, lower than that found in previous studies [8,9,10,11,12], may be partly attributed to the shorter duration of the intervention, regarding the evaluation of a single cycle of PDSA, in comparison to repeated cycles. Of note, the increased temperature during hospitalization, coupled with the reduced number of mild/moderate hypothermia episodes, highlighted the impact of the bundle in avoiding thermal deviations during the first days of life.

The present study reinforces the message previously highlighted by the work of Wotango et al [12] in a low-resource setting regarding the importance of temperature management in the immediate postpartum period and extends it to the days following admission to the NSCU and KMC room. This study also has some limitations that should be considered by the reader. First, the limited time span of the study period was driven by the need for a prompt assessment of the impact of the intervention, but also restricted the sample size and the chance to draw robust conclusions. Hence, further developments may focus on evaluating long-term impact and repeating the PDSA cycle to strengthen the current findings. Second, the single-center design limits the generalizability of the findings to similar settings. Third, the retrospective data collection limited the availability of some information, such as staff acceptability and compliance. Future studies may benefit from including data on the acceptance and implementation of measures by the staff and caregivers, which can provide useful information for improving the implementation of the bundle.

## 6. Conclusions

Within this study’s limitations, our findings provide useful information to clinicians and stakeholders about the importance and feasibility of thermal control in the postnatal period, which is not limited to temperature management immediately after birth but extended to the entire duration of hospitalization.

## Figures and Tables

**Table 1:** Bundled interventions to prevent neonatal hypothermia implemented in March 2024.

Area of Intervention	Action
**Training activity**	- Hypothermia prevention for delivery room staff. - Hypothermia prevention for NSCU staff. - Hypothermia prevention for students attending the ward. - KMC for mothers hosted in Kangaroo room.
**Delivery room**	The following procedures were implemented after the training course: - Placing a door at the newborn perinatal assistance room and turning on an electric radiator at least 1 h before delivery. - Radiant warmer on for at least half an hour before delivery with 100% heater output. - Prewarmed towel. - Removal of wet linen and wrapping in a prewarmed towel. - Skin-to-skin contact in the first hour after birth for stable newborns. - Appropriate newborn clothing (warm clothes including cap, socks, mittens, and sweater). - Newborn wrapping with prewarmed cotton and swaddle covering with wool blanket before moving to NSCU.
**NSCU**	The following procedures were implemented after the training course: - Turn on the electric radiator if room temperature < 26 °C. - Keep doors and windows closed. - Cover all newborns with cap, socks, and also mittens in case of birth weight < 1500 g. - Encourage Kangaroo care in NSCU also for unstable babies. - Cover with a blanket all the babies that are not under the radiant warmer. - Check body temperature every 30–60 min until 36.5 °C after a hypothermia episode. Always write the body temperature on the vital signs charts and alert the next shift. - Change the diaper at least 3 times a day. - Put two electric warmers near the cradle during phototherapy. - Let the radiant warmer always be on and set the power % to ensure a body temperature between 36.5 and 37.5 °C.
**Kangaroo Mother Care (KMC) room**	- Ensure KMC for all babies under 2000 g. - Ensure all newborns in KMC wear a hat and are covered with a blanket. - After managing hypothermia, check body temperature every 30–60 min until it reaches 36.5 °C. Always write the body temperature on the vital signs charts and alert the next shift. - Change the diaper at least 3 times a day.

**Table 2:** Characteristics of eligible neonates admitted to the NSCU in pre- and post-intervention periods.

	Pre-Intervention Period (n = 117)	Post-Intervention Period (n = 87)	*p*-Value
Mode of delivery:			0.36
Spontaneous vaginal delivery	63 (54%)	52 (60%)	
Assisted vaginal delivery	6 (5%)	7 (8%)	
Cesarean section	48 (41%)	28 (32%)	
Males	73 (62%)	46 (53%)	0.22
Birth weight:			0.43
≥2500 g	75 (64%)	62 (71%)	
1500–2499 g	35 (30%)	19 (22%)	
≤1499 g	7 (6%)	6 (7%)	
Respiratory distress	66 (56%)	50 (57%)	0.99
Jaundice	13 (11%)	4 (5%)	0.13
Early onset infections	26 (22%)	23 (26%)	0.59
Perinatal asphyxia	8 (7%)	8 (9%)	0.72
Oxygen therapy	81 (69%)	62 (71%)	0.87
Oxygen therapy (hours)	16 (7–43)	12 (8–34)	0.50

Data summarized as n (%) or median (IQR).

**Table 3:** Outcome measures in pre- and post-intervention periods.

	Pre-Intervention Period (n = 117)	Post-Intervention Period (n = 87)	*p*-Value
Neonatal temperature at admission, °C	35.6 (35.1–36.2)	36.0 (35.4–36.6)	0.004
Hypothermia (<36.5 °C) at admission	94 (80%)	61 (70%)	0.13
Average temperature during hospitalization, °C	36.3 (36.0–36.4)	36.7 (35.6–36.8)	<0.0001
Number of mild hypothermia (36–36.4 °C) episodes per day during hospitalization	1.0 (0.5–1.3)	0.5 (0.0–1.0)	<0.0001
Number of moderate hypothermia (32–35.9 °C) episodes per day during hospitalization	0.7 (0.3–1.0)	0.2 (0.0–0.5)	<0.0001
Number of severe hypothermia (<32 °C) episodes per day during hospitalization	Nil	Nil	-
Number of temperature checks per day during hospitalization	3.0 (2.5–3.2)	3.0 (2.7–3.3)	0.17
Mortality	5 (4%)	6 (7%)	0.61

Data summarized as n (%) or median (IQR).

## Data Availability

The raw data supporting the conclusions of this article will be made available by the authors on request.

## List of Abbreviations

HICs	High-income countries
NSCU	Neonatal Special Care Unit
WHO	World Health Organization
QI	Quality improvement
NICU	Neonatal Intensive Care Unit
KMC room	Kangaroo Mother Care room
PDSA cycle	Plan–Do–Study–Act cycle
